# FACTOR ANALYSIS OF CORNELL SCALE FOR DEPRESSION IN DEMENTIA AMONG ASSISTED LIVING RESIDENTS

**DOI:** 10.1093/geroni/igac059.1885

**Published:** 2022-12-20

**Authors:** Rachel McPherson, Barbara Resnick

**Affiliations:** University of Maryland Baltimore and Baltimore County, Catonsvile, Maryland, United States; University of Maryland, Baltimore, Maryland, United States

## Abstract

The Cornell Scale for Depression in Dementia (CSDD) was developed to measure depressive symptoms among older adults with dementia. The psychometric qualities of the CSDD have been inconsistent regarding the factor structure, with some studies showing a four-factor model and others a five-factor model. The purpose of this study was to test the factor structure of the CSDD as a measure of depression among a sample of assisted living residents. It was hypothesized that a four-factor version of the CSDD would provide a better fit than a five-factor version of the CSDD. The present study used baseline data from the Function-Focused Care for Assisted Living Using the Evidence Integration Triangle (FFC-AL-EIT) intervention study. A total of 511 residents from 85 assisted living facilities were included in the analyses. Confirmatory factor analyses were conducted to examine the factor structure and a chi-square difference test was conducted to compare model fit. Three items were removed from both models due to small factor loadings. The chi-square difference test indicated that the five-factor model fit the data significantly better (χ2 = 796.08, ∆χ2= 22.86, ∆df = 4, p < .001) than the four-factor model, although both the five-factor and four-factor models produced very poor model fits. These findings may be due to the fact that the CSDD relies on information from caregivers, and the measure might benefit from including observational signs of depression. Future work should examine other factors or items that belong in a depression measure among assisted living residents.

